# The Importance of Early Treatment of Thyroid Hyperplasia

**Published:** 1914-12

**Authors:** R. P. Higgins

**Affiliations:** Cortland, N. Y.


### The Importance of Early Treatment of Thyroid Hyperplasia.

DR. R. P. HIGGINS, Cortland, N. Y.

The recent development of surgery of the thyroid gland and the brilliant results obtained by dexterous operators have for a time obscured the medical treatment of hyperplasia of the thyroid. It is particularly in the early stages of thyroid disease that medical treatment is of avail, before marked organic changes have taken place in the gland. The early symptoms are suggestive of functional disturbance. When these are unrelieved it may be assumed that organic changes are not long postponed. The surgeon's knife will be needed in many cases to cut the vicious circle when once established and new formations of tissue with resulting persistent hyperthyroidism threaten the existence of the patient and make life almost unbearable.

Of all the glands having to do with the vital processes of the body the thyroid is most accessible to examination but even at the present its exact action is but poorly understood. Its blood supply is very rich and the blood vessels and lymphatics are remarkable for their large relative size and their frequent and rich anastamoses. The gland can be demonstrated to change in size by alteration in its blood supply. This may be due to an interference in the return of the venous flow such as improper use of the voice, shouting, by the lifting or carrying of heavy loads or by the obstruction of tight collars or bands about the neck. There is a close relationship of the size of the gland to the activity of the genital organs. A visible enlargement may be demonstrated at the time of puberty, during pregnancy, at each menstruation, and after coitus.

The function of the thyroid seems to be confined to the production of its secretion, but of the actual nature of the secretion but little is known. It is supposed that a substance of albuminoid construction is elaborated and that this is sometimes stored as colloid, but after some chemical reaction with iodine it passes into the general circulation in the form spoken of as iodothyrin or some allied preparation. It is to the iodine content that the various thyroid preparations owe their efficacy. The function of this internal secretion seems to be that of an accelerator or sensitizer to the central nervous system and through that to the general system. It is probable moreover that the thyroid secretion acts more directly as a sensitizer to other organs and tissues of the body, certain it is that it acts as an accelerator to protoplasmic combustion.

As Crile aptly remarks "At will, then, through diminished, normal or excessive administration of thyroid secretion we may produce an adynamic, a normal, or an excessively dynamic state. By the thyroid influence, the brain thresholds are lowered and life becomes exquisite; without its influence the brain becomes a globe of relatively inert substance. Excessive doses of iodine alone cause most of the symptoms of Graves' disease. . . . The essential pathology of shock is identical whatever the cause. If however instead of an intense overwhelming activation, the kinetic system is continuously or intermittently overstimulated through a considerable period of time, as long as each of the links in the kinetic takes the strain equally the result will be excessive energy conversion, excessive work done; but usually under stress, some one link in the chain is unable to take the strain and then the evenly balanced work of the several organs of the kinetic system is disturbed. If the brain cannot endure the strain, then neurasthenia, nerve exhaustion, or even insanity follows. If the thyroid cannot endure the strain it undergoes hyperplasia, which in turn may result in a colloid goiter or in exophthalmic goiter. If the suprarenals cannot endure the strain, cardiovascular disease may develop. If the liver cannot take the strain then death from acute acidosis may follow, or if the neutralizing effect of the liver is only partially lost, then the acidity may cause Bright's Disease. Over activation of the kinetic system may cause glycosuria and diabetes."

From clinical findings it is evident that at the onset of thyroid disease we first get exhaustion of the gland, either from the effect of a prolonged nervous or mental strain, or from the effect of autointoxication or some irritant poison of disease working on the system. The close connection of the thyroid gland with the genital organs in women may explain the reason why perversion of thyroid function falls more frequently on women than on men. The first symptoms in nearly all cases of thyroid disease are those of thyroid exhaustion and deficiency of thyroid secretion.

In this first stage there is generally a history of a prolonged nervous strain, anxiety or over excitement. The patient complains of a feeling of weariness and mental lassitude and is easily fatigued. There may be headaches and constipation and disturbances of digestion. The blood pressure is slightly lower than normal and generally ranges below 120mm of mercury. The condition is most apt to develop during puberty, pregnancy, or the menopause. So far the symptoms may be said to be those of a typical case of neurasthenia but in addition we get the enlargement of the thyroid gland either visible or to be demonstrated by palpation. The enlargement is generally of the whole gland, is soft at first and is generally symmetrical. If the patient is put under the influence of rest and correction of the deleterious influences at work we may get the disease arrested at this point. Should however the disease be allowed to progress we get a compensatory hypertrophy in the thyroid with symptoms developing of hyperthyroidism. If you will analyze the histories of your cases of exopthalmic goiter you will find that almost invariably they give at the onset symptoms of deficiency of thyroid secretion followed after a time by pathological increase of thyroid secretion. The prolonged increased action of the thyroid brings about the tachycardia, the nervousness, and tremor with the exophthalmos and the enlargement of the thyroid making up the picture of the disease.

It is obvious that when we have gotten a hypertrophied action of the thyroid and have definite organic changes in its substance it is not possible to retrace by treatment all the steps which have led up to this condition and only some form of operation on the thyroid will help our patient, but why let our patients drift to this point? Early intelligent treatment of many of these cases of thyroid hyperplasia will arrest and cure the trouble in its incipiency, when if it is let to run to organic changes in the thyroid and associated organs, treatment by drugs does not do so much good. It is true that medical treatment to be of assistance must be begun early and that many of our patients come to us too late for it to be of avail. But just as in the cure of cancer we must make an early diagnosis, so we must do it in thyroid disease and not let our patients drift along from one physician to another and steadily getting worse with an indefinite diagnosis of neurasthenia, hysteria or anaemia without considering the possibilities of thyroid disturbance.

In the development of the condition of hyperthyroidism from the condition of thyroid exhaustion or hypothyroidism the goiter which at first was rather soft becomes somewhat more firm in consistence. The most characteristic symptoms is the pulse which in the first stage was either normal or slower than normal, and now becomes increased. The blood pressure slightly raises. The nervous system which was in a state of mental lethargy becomes overactive and excitable and often a fine tremor of the hands becomes manifest. The muscular reflexes become exaggerated. There is an irritability of the nervous system. There is often the development of a slight fever. In about 25% of cases exophthalmos develops and when it appears it is indicative of a more serious condition. After the stage of hyperthyroidism has persisted for some time, organic changes may take place in the heart and kidneys which may mask the original cause of the trouble. There is generally a rise of blood pressure and either a hypertrophy or dilation of the heart or a combination of both, and occasionally the development of a nephritis.

Very occasionally hyperthyroidism may develop without the presence of a demonstrable goiter. These conditions present the tachycardia, the nervous manifestations and even the exophthalmos without the enlargement of the thyroid becoming manifest. These conditions are generally of unusual severity and do not as readily respond to treatment as in cases with the presence of an enlargement of the thyroid. Microscopic examination however of the portions of the excised gland shows that small portions of the gland have already undergone the pathological change characteristic of the disease.

Treatment of all these conditions to be effective must be directed towards the exciting causes of the disorder. The greatest care should be given to the taking of the history and the examination to find what exciting causes of long duration have been at work. If the person has been under an undue nervous or mental strain she should be immediately put to bed and removed as far as possible from any nervous excitement of whatever nature. The most intractable case of hyperthyroidism with which I ever had to deal was a woman who could not or would not relax in her strenuous overactivity. As a matter of fact the enforced rest after any form of operation that may be undertaken in many of these cases may be as efficacious as the primary effect of the operation.

Many times the long continued absorption of intestinal toxins or the toxins of disease have been the exciting cause of the thyroid disturbance and measures should be taken to guard against this. As a matter of fact in the early stages of the disease rest of mind and body together with relief of the exciting causes may serve to bring about a cure without any further measures. We should make every effort to break up the vicious circle that has been established.

As the early stages of thyroid disease is generally accompanied by a lessened amount of thyroid secretion it is important to make a diagnosis as to a probable amount of thyroid secretion circulating through the body. At the present time we have no means of definitely measuring the amount of secretion the thyroid is elaborating but we have to depend mainly on the symptomatology of the case. If the patient is dull, lethargic, easily fatigued, with a low blood pressure together with slight thyroid enlargement we can conclude that we are dealing with a condition of lessened thyroid secretion. Clinical evidence is abundant that the administration of thyroid extract in these conditions of hypothyroidism is almost specific in its effect. The usual dose is from 3 to 5 grains of a reliable extract three times a day.

In the other, type of cases where in addition to our thyroid enlargement we have symptoms of heightened activity of the gland such as nervous irritability, increase of muscle reflexes, increase of arterial pressure, gradual development of tachycardia with associated cardiac and nephritic lesions and in most severe cases exophthalmos. This class of cases is suffering from an excess of thyroid secretion thrown into the circulation and the treatment is manifestly different from the first class of cases. Absolute rest of mind and body is most essential in these cases. I have found that a diet from which meat, tea and coffee and condiments are eliminated has produced the best results. Alcohol and tobacco should be forbidden.

From time to time various writers have come forward with a specific serum to be used in the treatment of conditions of hyperthyroidism. In a recent communication Rogers, who with Beebe introduced one of the most popularly used form of antiserum, limits its activity materially. He says:

''At the outset of my experience the antithyroid serum seemed a simple solution of the problems presented by hyperthyroid symptoms. The serum is made by injecting rabbits or sheep at intervals for about six weeks with proteins obtained from the human thyroid gland. It was designed to inhibit the excessive activity of the thyroid epithelium and to neutralize its product. But at the end of a few years the results showed only 15 percent or 20 percent of perfect cures, and some 50 percent of more or less marked development. There was a considerable percent of failures and relapses, and among these a morality of about 8 percent. These statistics included all types of the abnormal process and I had not learned to differentiate it into the stages which seem to represent both its degree of severity and the other organs which may be primarily or secondarily involved.''

''The mortality on untreated cases is variously estimated as between 5 percent and 10 percent as a more or less direct result of the hyperthyroidism and therefore, the mortality of cases treated by the antiserum cannot be regarded as caused by it. The serum, may however be injurious, if its administration is continued in spite of manifest intensification of the symptoms. But this antiserum presents obvious and practically insurmountable difficulties, not only in the limitation of the supply, but in the limitation of its usage to certain types of the disease. It is extremely beneficial in the early and uncomplicated cases of hyperthyroidism and has proved almost a specific in the rare instances of early acute toxaemic hyperthyroidism. But it is beneficial or curative for only a few of those who show the symptoms of exophthalmic goitre, or the presumably later and more severe and complicated thyroid fatigue.''

''The majority of patients who suffer from hyperthyroidism, as ordinarily encountered, have passed far beyond the earlier and more easily relieved conditions, and it is, therefore, necessary to supplement or supplant entirely the antiserum by measures which, with the prevailing conceptions of the disease, are practically more capable of general application.''

The use of iodides and iodine preparations in this class of cases while they may serve to decrease the size of the thyroid gland are almost certain to intensify the associated symptoms.

Forcheimer advises in the internal treatment of this class of cases the internal administration of quinine hydrobromide either alone or in connection with ergotin. He advises it administered in the form of gelatine coated pills composed of 5 grains of quinine hydrobromide and 1 grain of ergotin and administered four times a day and as the case improves the number of pills may be dropped to 2 or 3 times a day. It is difficult to explain the exact action of this preparation but it is probably due to cardiac tonic and vaso constrictor properties, and there may be a more direct effect on the toxaemia. The results he has attained by this method are certainly as good as have been attained by any other method of internal treatment.

In many cases the calcium salts are beneficial for their tonic effect on the heart and because in this disease there has been demonstrated an increased elimination of calcium in the urine. They may be given as the calcium lactate in doses of 5 to 7 grains administered three times a day. The glycerophosphates of lime and soda are beneficial in this class of cases as in addition to the effect of the calcium we get the tonic effect of the phosphorus.

In connection with the treatment as outlined above great care should be taken to keep the organs of elimination acting freely. Many experienced clinicians have come to the conclusion that the best remedy in conditions of hyperthroidism is to maintain a free condition of the bowels and kidneys and to prevent autointoxication. Only the milder salines should be used such as phosphate of sodium. Too vigorous purging tends to deplete the system and lower the body vitality. In addition the patient should be urged to drink large quantities of water and in regions where goiter is endemic the water should be boiled or distilled.

Local external treatment of the thyroid gland certainly is of some assistance in relieving the trouble. The old method of treatment was by the application of tincture of iodine and this method certainly was productive of some results but it has the disadvantage of being disagreeable and of at times setting up a dermatitis. A non-irritating iodine absorbable ointment or oily solution such as iodo vasogen or iodine petrogen have seemed to be of considerable benefit. In the application of external measures to the skin over the thyroid care should be taken not to manipulate the thyroid too roughly as it is possible to milk some of the secretion into the general system in this way.

Certain writers have advised to inject into the substance of the gland of irritating solutions such as a 5% aqueous solution of pure carbolic acid, various iodine solutions and the like. This method of treatment has been highly commended by experienced observers but personally I have never inclined to its use.

The use of the X-ray undoubtedly in the hands of a careful operator may reduce the size of a hyperthyroid gland and may decrease its functional activity. If there is any possibility that the case may later have to be operated, the X-rays should not be used as they tend to produce adhesions in the tissues rendering an operation more difficult. Other forms of electricity that have been advised are of value mainly for their suggestive effects.

Certain cases of thyroid disease should be immediately referred to the surgeon. Kocher gives the following list of cases which should be operated:

"(1) Internal treatment is useless in struma nodosa with nodules in process of secondary degeneration. Degenerative nodules can also be recognized directly by the changes in their consistency. Thus, all colloidal degenerated nodules (struma gelatinosa), as well as fibrous calcareous, hemorrhagic, and cystic nodular goiter, must be at once turned over for operative treatment.

(2) Diffuse colloidal tumors that have resisted several brief periods of iodine medication must be referred to the surgeon, especially if they have already given rise to functional disturbances.

(3) All goiters that cause pronounced pressure symptoms must be treated by operation.

(4) The same is true of those which produce cardiac symptoms and (5) of goiters that are abnormally situated, especially struma profunda and intrathoracic, which are very dangerous if the tumor continues to grow.

(6) If a goiter develops suddenly and grows very rapidly and if the shape and consistency are unusual it must be treated by operation regardless of the patients age.

(7) A goiter showing sentitiveness on pressure, especially if it causes spontaneous pain, must be referred to the surgeon."

It is evident that the above mentioned classes of cases are the more advanced cases and those which may serve as a basis for cancerous degeneration. In cases of this nature time should not be wasted in internal treatment. But in the class of cases of alteration in the amount of thyroid secretion and symmetric enlargement of the thyroid the greater percentage can be cured or relieved by appropriate internal medication.

